# AI‐Powered Speech Analysis: Automating Transcription, Embeddings, and Deep Learning for Early Alzheimer's Detection

**DOI:** 10.1002/alz70856_107467

**Published:** 2026-01-09

**Authors:** Daniel Zhou, Zhen Qian

**Affiliations:** ^1^ Skyline High School, Ann Arbor, MI, USA; ^2^ University of Michigan, Ann Arbor, MI, USA

## Abstract

**Background:**

Spontaneous speech is a promising, non‐invasive, cost‐effective biomarker. LLM vector embeddings capture semantic and contextual patterns. This study transcribed audio, generated embeddings, and trained machine learning models to classify AD patients versus healthy controls.

**Method:**

We used audio files from the Alzheimer's Dementia Recognition through Spontaneous Speech (ADReSS) 2020 Challenge dataset, with 108 training participants (54 AD, 54 healthy) and 48 testing participants, all describing the Cookie Theft picture. We did linguistic analysis for word frequency and speech disfluency. Next, we evaluated two commercial audio‐to‐text APIs (OpenAI Whisper vs AssemblyAI) to build a more automated and scalable classification pipeline. We used two OpenAI's new embedding models to generate embedding vectors. Lastly we built three classification models: Support Vector Machine (SVC), Logistic Regression (LR) and Random Forest (RF) and compared model performance.

**Result:**

We can find a higher frequency of filler words and fewer diversity of vocabulary in the word cloud of Alzheimer's. The classification model's ROC values are significantly improved when the LLM vector embedding is used, compared to the model based solely on linguistic features. Combining vector embeddings with linguistic features only resulted in marginal improvements. We used both the OpenAI Whisper and the AssemblyAI to generate audio transcriptions. We listed detailed metrics for the classification model trained with embedding vectors combined with linguistic features. The Support Vector Machine (SVM) model out‐performed the Logistic Regression(LR) and Random Forest(RF) models with 0.84 in Accuracy, 0.83 in Precision.

**Conclusion:**

We have built an AI‐based pipeline to transform spontaneous speech recordings into Large Language Model vector embeddings, and compared performance of different machine learning models for Alzheimer's classification. The project source code is hosted on GitHub as an open source project: https://github.com/dzhou08/embedding_AZ In the future, we will integrate this pipeline into a web application as a free service. We will also look into possibilities of combining speech embeddings with other multimodal biomarker data to further enhance the diagnostic accuracy. With the rapid advance of AI and sensor technologies, multimodal frameworks will likely become standard in dementia care in the near future.

